# Arterial pathophysiology and comparison of two devices for pulse wave velocity assessment in elderly men: the British regional heart study

**DOI:** 10.1136/openhrt-2017-000645

**Published:** 2017-12-17

**Authors:** Elizabeth A Ellins, Kirsten E Smith, Lucy T Lennon, Olia Papacosta, S Goya Wannamethee, Peter H Whincup, Julian P Halcox

**Affiliations:** 1 Institute of Life Sciences, Swansea University Medical School, Swansea, UK; 2 UCL Department of Primary Care & Population Health, UCL Medical School, London, UK; 3 Division of Population Health Sciences and Education, Population Health Research Institute, St George’s University of London, London, UK

**Keywords:** cardiovasclar examination, atherosclerosis, peripheral vascular disease

## Abstract

**Objective:**

Vascular disease is highly prevalent in the elderly. This study aimed to evaluate arterial phenotype in elderly men and compare carotid–femoral pulse wave velocity (cfPWV) assessed by two techniques (Sphygmocor (S)and Vicorder (V)).

**Methods:**

1722 men (72–92 years), participants in the British Regional Heart Study, underwent ultrasound assessment of carotid intima–media thickness (cIMT), carotid distensibility coefficient and presence of carotid plaque. cfPWV and ankle brachial pressure index (ABPI) were also assessed. 123 men returned for between visit reproducibility assessments.

**Results:**

Good reproducibility was demonstrated in all measures (Gwet’s agreement=0.8 for plaque, intraclass correlation=0.65 for ABPI and coefficient of variation <13% for all other measures). Measurements were obtained in >90% of men for all measures except cfPWV(S) and ABPI. In 1122 men with both cfPWV(V) and cfPWV(S) data, cfPWV(S) was greater than cfPWV(V) (mean difference=0.23,95%CI 0.10 to 0.37 m/s). cfPWV(V) was higher at low cfPWV values and cfPWV(S) was higher at high cfPWV values. Correlation of V transit time (TT) against S carotid and femoral TT demonstrated that the slope of the regression line for femoral TT was steeper than for carotid TT, resulting in a proportionally greater subtraction of carotid TT from femoral TT at higher PWVs.

**Conclusions:**

Reproducible, satisfactory quality non-invasive measurements of vascular phenotype were obtainable in a large proportion of elderly men. The discrepancy in results between the two PWV measures may partly be due to the differential impact of subtracting carotid TT when deriving cfPWV(S) across the clinical PWV range.

**Key questions**

**What is already known about this subject?**

Non-invasive measures of vascular structure and function are associated with risk of cardiovascular disease and mortality.

**What does this study add?**

- Demonstrates that good quality non-invasive vascular measures are achievable in elderly men.
- It is the largest comparison of Vicorder and Sphygmocor techniques for assessment of pulse wave velocity and the first in an elderly cohort.

**How might this impact on clinical practice?**

Accurate assessment of PWV is essential for its use in cardiovascular risk assessment of patients with hypertension. Identification of differences in pulse wave velocity values due to method of assessment are import as they may lead to incorrect risk classification and treatment of these patients.

## Introduction

Ageing is a strong determinant of cardiovascular disease (CVD) which accounts for about a third of deaths in over 65 years of age both in the USA and the UK.^1 2^ Carotid intima–media thickness (cIMT), aortic carotid-femoral pulse wave velocity (cfPWV) and ankle brachial pressure index (ABPI) are measures of vascular structure and function which increase with age and are independently associated with increased risk of CVD and mortality.^3–7^ However, only limited data in the very elderly describe the relationship between these measures of vascular pathophysiology and CVD outcomes in the context of lifetime risk factor profile. Furthermore, the ease of obtaining reliable data and the relative ability of these techniques to predict outcome in an elderly population is unclear, either as individual measures or in combination.

Two methods are widely used for assessing aortic PWV. The Sphygmocor (Atcormedical) uses applanation tonometry to capture sequential ECG-gated pulse pressure waveforms at the carotid and femoral arteries.^8^ However, this technique has some practical challenges and can be operator dependent. The Vicorder uses a volume displacement technique to assess pulse pressure waveforms simultaneously using inflatable cuffs positioned at the carotid and femoral arteries.^9^ Recent comparisons have shown agreement between the Sphygmocor and Vicorder methods in both children and adults (aged 19–92 years) in small populations of up to 122 participants, but have identified a potential bias between techniques.^9–13^ However, there has been no large systematic comparison of the two methods or comparative studies in elderly subjects, who are more likely to have stiffer arteries with many in the range associated with high future cardiovascular risk. It is important to identify differences in measurements between techniques as they may have potential implications for the prognosis and clinical management of elderly participants.

In this paper, we present a range of common non-invasive vascular parameters in a large cohort of older men. We also provide an assessment of the success of application and comparability of two methods of measuring cfPWV in a population of elderly men.

## Methods

The 3137 survivors of the British Regional Heart Study were invited to undergo vascular assessment between 2010 and 2012, either at a local clinic or, if not able to attend, in their own home where they underwent a limited assessment. Participants provided informed written consent to the study, in accordance with the Declaration of Helsinki. A fuller description of the cohort and methods can be found in the online supplementary material.

10.1136/openhrt-2017-000645.supp1 Supplementary file 1

### Blood pressure

Seated blood pressure was recorded from the right arm with an automated sphygmomanometer (Omron HEM 907, Japan) using an appropriately sized cuff. Two measurements were made 1 min apart; the results were averaged.

### Pulse wave velocity

Two techniques were used to assess cfPWV: applanation tonometry (Sphgymocor (S), Atcormedical, Australia) and an oscillometric cuff-based technique (Vicorder (V), Skidmore Medical, UK). Measurements were taken with the participant supine, with their torso at approximately 30°. cfPWV(S) was assessed first followed by cfPWV(V).

### Carotid artery ultrasound measurements

Carotid arteries were imaged using the Z.One Ultra ultrasound system (Zonare Medical Systems, Mountain View, California, USA) with a 5–10-mHz linear probe. Recordings were taken in DICOM format for later offline analysis.

#### cIMT and distensibility coefficient

Peak systolic and end-diastolic carotid artery diameter and cIMT (the distance between the leading edge of the intima and the media–adventitia interface) were measured from longitudinal images using Carotid Analyser software (Medical Imaging Applications, Iowa City, Iowa, USA). Distensibility coefficient (DC) for each artery was then calculated from the distension and ipsilateral blood pressure and then averaged to give overall DC.^14^

#### Plaque identification

Carotid artery ultrasound scans were assessed for the presence of atherosclerotic plaques by five trained observers, using either the Z.One Ultra ultrasound system or Microdicom software (Microdicom, Bulgaria). Carotid plaques were classified as a focal area of IMT ≥1.2 mm at its thickest point or ≥50% of the adjacent IMT.^15 16^

### Ankle Brachial Pressure Index

ABPI measurements were taken sequentially on both right and left sides, using the Vicorder device (Skidmore Medical, UK).

### Reproducibility assessment

Between visit reproducibility of vascular measurements and reproducibility of ultrasound analysis were formally evaluated.

### Classification of cardiovascular risk status by cfPWV(V) and cfPWV(S) results according to expert consensus thresholds for clinical practice

Participants cardiovascular risk status was classified according to their cfPWV(V) and cfPWV(S) results using cut-off values suggested by a European guidelines paper (cut-off 12 m/s) and a later European expert consensus paper (cut-off 10 m/s).^17 18^

### Statistical data analysis

Analyses were performed using SPSS software (V.20.0, SPSS, Chicago, Illinois, USA). Data are presented as mean±SD or 95% CI unless otherwise stated. Variability of the vascular measures was assessed by coefficient of variation (CV%). Exceptions to this were ABPI (intraclass correlation (ICC)) and identification of the presence of plaque (Gwet’s agreement coefficient 1 (AC1), which allows assessment of inter-rater reliability while overcoming statistical issues associated with the alternative Cohen’s Kappa method.^19^ Age group analyses were performed using analysis of variance with Bonferroni’s post hoc test or Χ^2^ as appropriate.

Differences between the two PWV methods were investigated using paired t-tests and Bland-Altman plots.^20^ Correlations were examined using Pearson coefficients. Linear regression was used to investigate the relationship between difference in PWV and the mean PWV of the two methods; models were adjusted for age and systolic and diastolic blood pressure, heart rate, height, weight and body mass index (BMI) as individual variables. A final model included age, diastolic blood pressure, heart rate and BMI simultaneously entered into the model.

## Results

### Characteristics of cohort

A total of 1722 men (mean age 78.5±4.7 years) attended the vascular clinics (n=1636) or were seen at home (n=86). The mean anthropometric measures for the cohort were as follows: height 1.71±0.6 m, weight 79.8±12.7 kg and BMI 27.2±3.8 kg/m^2^. Mean systolic and diastolic blood pressures were 145±19 and 76±12 mm Hg, respectively. Measurements were attempted in all men except for 28 in whom cfPWV(S) and ABPI were not evaluated (protocol reduction for staff sickness). Table 1 shows the number of participants (assessed in clinic and at home) in whom successful measurements were taken with mean values. cIMT, presence or absence of plaque, DC and cfPWV(V) were successfully obtained in over 90% of participants. Details regarding missing data for ABPI and cfPWV measures are described in the online supplementary material.

**Table 1:** Number and percentage of men in which each measurement of non-invasive arterial parameter was achieved with mean values for each measure and number (percentage) of men in whom plaque was seen plus between visit reproducibility analyses

Between visit reproducibility						
Measurement	n (% of whole cohort)	Value*	n	Visit 1 (mean±SD)	Visit 2 (mean±SD)	CV%
cfPWV(S) (m/s)†	1179 (73%)	10.3±2.6	83	9.9±2.6	9.9±2.6	9.5
cfPWV(V) (m/s)	1577 (92%)	10.2±1.7	112	10.2±1.5	10.3±1.5	5.1
DC (x10^−3^ kPa^−1^)	1687 (98%)	12.3±4.2	116	12.2±3.8	12.7±4.6	12.0
Distension (mm)	1703 (99%)	0.41±0.13	120	0.41±0.12	0.41±0.13	10.3
cIMT (mm)	1696 (98%)	0.81±0.16	120	0.79±0.14	0.75±0.13	7.8
Plaque	1717 (99%)	1444 (84%)	123	76%	88%	0.8‡
ABPI	1369 (80%)	1.13±0.14	92	1.13±0.11	1.18±0.11	0.65§

Results for ultrasound analysis variables show interobserver and mean intraobserver (for cIMT and distension mean of two observers for plaque mean of five observers) reproducibility for each measure.

*Data presented as mean±SD and number and percent with plaque.

†Not assessed during home visits, percentage achieved calculated from the 1634 who attended clinic visits.

‡Gwet’s Agreement Coefficient measured reproducibility in plaque identification.

§ICC measured agreement in ABPI. Data are mean±SD or percentage presence.

ABPI, ankle brachial pressure index; cfPWV, carotid–femoral pulse wave velocity; cIMT, carotid intima-media thickness; CV, coefficient of variation; DC, distensibility coefficient; ICC, intraclass correlation; n, number of subjects; S, Sphygmocor; V, Vicorder.

Between visit reproducibility results were acceptable for all measurements (table 1). Inter- and intra-observer reproducibility results for the offline analysis for measuring cIMT (inter n=109 CV%=7.1%, intra n=30 CV%=5.1%), distension (inter n=109 CV%=9.2, intra n=30 CV%=11.9%) and presence of plaque (inter n=20, AC1=0.9; intra n=40, AC1=0.8) were also acceptable. cIMT, prevalence of plaque and cfPWV all increased with age (online supplementary figure 1). DC and ABPI both decreased with increasing age (online supplementary figure 1). A total of 415 men had cIMT ≥0.9 mm, of whom only 36 did not have plaque. Plaque was seen in 72% of right and 73% of left carotid arteries. Of the 1369 with ABPI readings, 119 (8.7%) had ABPI below the clinical threshold for diagnosis of peripheral arterial disease (PAD) (<0.9).

### Comparison of PWV measured with Sphygmocor and Vicorder

A comparison of the Vicorder and Sphygmocor methods used to assess cfPWV was undertaken in the 1606 men who had cfPWV assessed by both techniques (28 of the 1634 did not have cfPWV(S) attempted in clinic due to staff illness; cfPWV(S) was not assessed during home visits). Good quality cfPWV readings were achieved with both methods in 1122 participants (characteristics in online supplementary table 1). Men with cfPWV(V) readings but no cfPWV(S) data (n=359) were older, with lower systolic blood pressure, a higher heart rate and greater cfPWV(V) than those participants with cfPWV measurable using both methods (online supplementary table 1). There were no significant differences in those parameters between the small proportion of men with cfPWV(S) but not cfPWV(V) readings (n=57) (online supplementary table 1).

**Figure 1:**
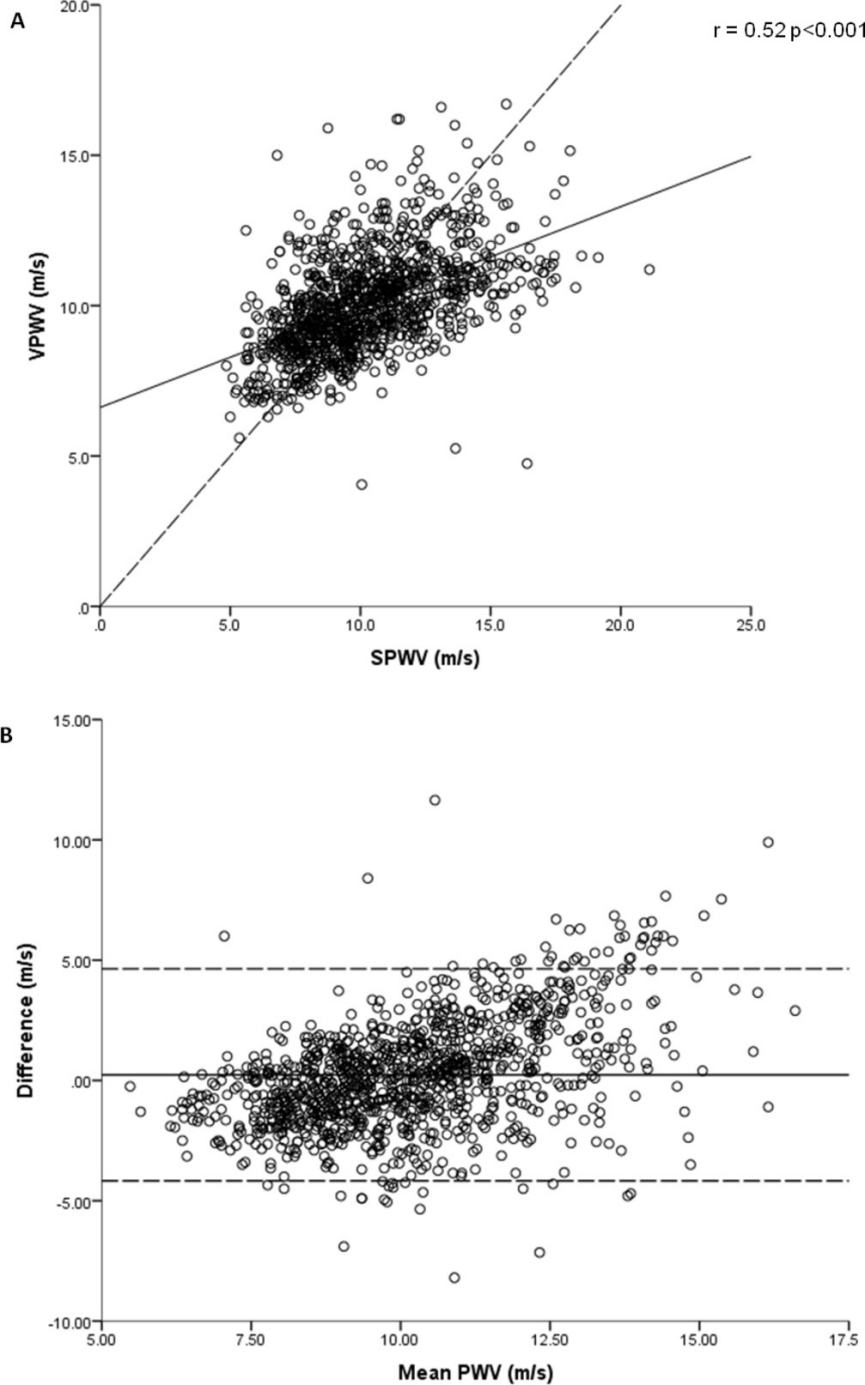
(A) Correlation between SPWV and VPWV, dashed line = line of unity solid line = regression line (B) Bland-Altman plot of agreement between SPWV and VPWV dashed line = 95% CI solid line = mean difference. SPWV, Sphygmocor pulse wave velocity; VPWV, Vicorder pulse wave velocity.

Table 2 shows the mean cfPWV values in the 1122 participants with results obtainable by both methods. cfPWV(V) was only moderately correlated with cfPWV(S) (r=0.52, P<0.001) with limits of agreement of −4.17 to 4.26 m/s (figure 1A). The mean difference in cfPWV between the two methods was 0.23 m/s (95% CI 0.10 to 0.37) across the range (4.1 to 21.1 m/s). Bland-Altman analyses demonstrated potential bias between the measures across the cfPWV distribution, with a tendency for higher cfPWV(S) readings than cfPWV(V) at higher cfPWVs and vice versa (figure 1B). Linear regression of the difference in cfPWV and the mean PWV of the two methods indicated that the difference between the two methods varied significantly across the PWV range (B=0.56, 95% CI 0.50 to 0.63, P<0.001).

**Table 2:** Mean cfPWV, path length, transit time and heart rate for the two methods of assessing cfPWV

n=1122	Sphygmocor	Vicorder	Diff	P value
cfPWV (m/s)	10.28±2.6	10.05±1.7	0.23 (0.10–0.37)	<0.001
Path length (mm)	423±37	693±44	−270 (−272 – −268)	<0.001
Transit time (ms)	43.89±10.31	70.76±11.70	−26.87 (−27.50– −26.24)	<0.001
Heart rate (bpm)	59.68±11.52	59.93±10.08	−0.26 (−0.69–0.18)	0.25

cfPWV, carotid–femoral pulse wave velocity; Diff, difference between Sphygmocor measures and Vicorder measures, calculated as Sphygmocor minus Vicorder.

#### Differences in path length

Taking different approaches to path length estimation for each technique according to the other manufacturers approach or current guideline recommendations for both Vicorder and Sphygmocor increased the difference in PWV between the measures but the bias remained (see the online supplementary material and supplementary figures 2 and 3).

#### Differences in transit time

**Figure 2:**
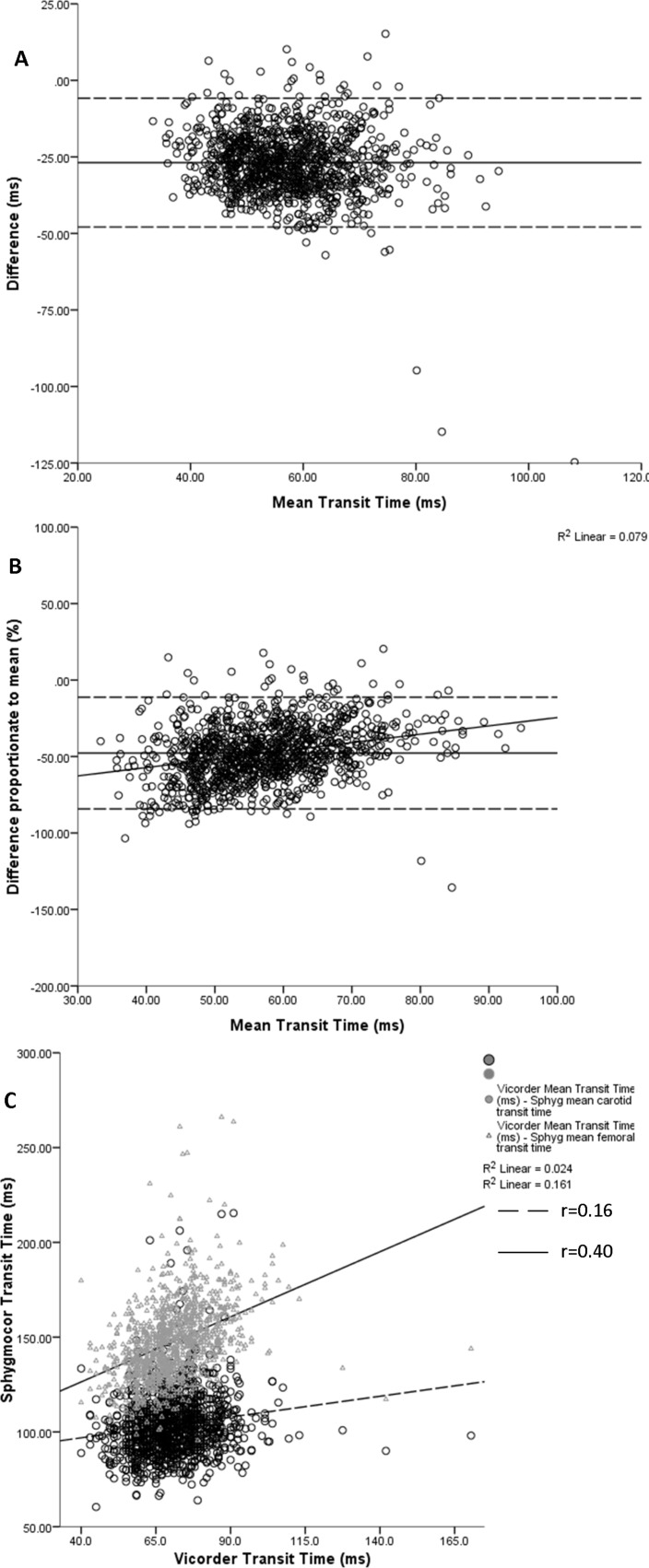
Bland-Altman plots of agreement between (A) Sphygmocor transit time and Vicorder transit time; (B), Sphygmocor transit time and Vicorder transit time with the difference between the two devices represented as a proportion of the mean transit time. (C) Association between Vicorder transit time and carotid and femoral transit time from the Sphygmocor.

We then investigated the potential impact of methodological differences in determination of transit time between the two methods. Transit times for the two methods were moderately correlated (r=0.53, P<0.001) but the Vicorder transit time was greater than that of the Sphygmocor, in keeping with the greater path length (table 2). Bland-Altman analysis of Sphygmocor versus Vicorder transit times showed systematic differences across the range, with Spyhgmocor being shorter than Vicorder (figure 2A). When the difference between Sphygmocor and Vicorder transit times was expressed as a proportion of the mean transit time, there was a greater proportional difference at shorter transit times favouring calculation of a relatively greater cfPWV(S) (figure 2B).

We were interested in whether the ‘subtraction’ method required for transit time calculation with Sphygmocor could result in a systematic difference and potential bias across the cfPWV range. We found a stronger correlation between femoral transit time and Vicorder transit time than between carotid transit time and Vicorder transit time (r=0.40 vs 0.16, respectively, figure 2C). Thus, as participants’ transit times shorten, a relatively greater proportion of carotid transit time will tend to be subtracted from the femoral transit time when deriving cfPWV(S), than that at longer transit times, resulting in progressively shorter transit times at faster cfPWV and relatively lengthened transit times at slower cfPWV.

#### Other factors

Linear regression was used to investigate whether other factors such as age, blood pressure or anthropometric measures accounted for the differences seen between the two techniques. Following adjustment for age the association between difference in cfPWV between the two methods and the mean cfPWV was still significant (B=0.59, 95%CI 0.53 to 0.66, P<0.001). The relationship remained following adjustment for age, diastolic blood pressure, heart rate and BMI (B=0.60, 95% CI 0.54 to 0.67, P<0.001) with age, diastolic blood pressure and BMI being significant predictors for the difference in PWV between the two methods (online supplementary table 2).

#### Identification of cardiovascular risk by PWV, according to recommended cut-off values

**Figure 3:**
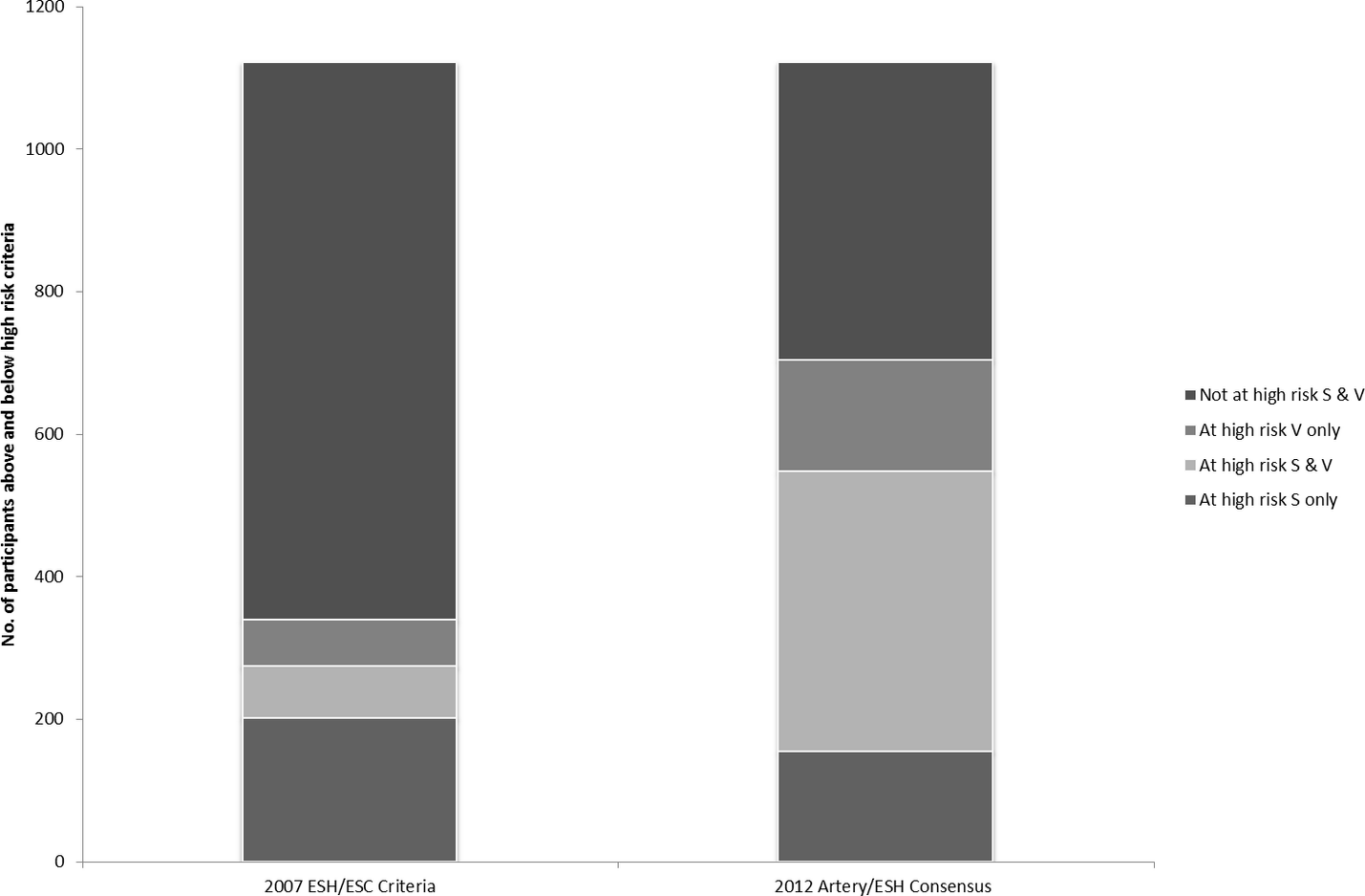
Comparison of 2007 European Society of Hypertension (ESH)/European Society of Cardiology criteria and 2012 Artery/ESH consensus to identifying ‘at risk’ groups from Sphygmocor (S) and Vicorder (V) PWV results. PWV, pulse wave velocity.

We investigated the effect of the difference in cfPWV results between the two methods, particularly at the higher end of the cfPWV range, on cardiovascular risk identification based on cfPWV values. Cut-off values of 12 and 10 m/s were used as suggested by the 2007 European hypertension guidelines and the 2012 European expert consensus paper, respectively.^17 18^ With a cut-off value of 12 m/s,^17^ 274 (24%) men were considered as being at increased cardiovascular risk according to their cfPWV(S) readings, with 138 (13%) according to their cfPWV(V) reading and only 72 (7%) according to both techniques (figure 3). Using the cut-off value of 10 m/s,^18^ 548 (49%) men were considered at increased risk using the Sphygmocor and 549 (49%) using the Vicorder; 393 (35%) men were classified at increased risk by both methods.

## Discussion

In this study we have shown that non-invasive characterisation of the arterial phenotype in a large population of elderly men is achievable; all vascular measures were reproducible and were strongly related to age.

We have also shown that cfPWV was greater on average when assessed using the Sphygmocor and that there was only moderate correlation between Vicorder and Sphygmocor techniques. Furthermore, a Bland-Altman plot showed that the bias between the two instruments differed according to absolute cfPWV, with the Vicorder tending to give lower measurements than Sphygmocor at the higher end of the PWV range and higher measurements at the lower end of the cfPWV range. Adjusting the way respective path lengths were calculated did not alter this bias.

### Cohort characteristics

Good quality data were obtained in >90% of elderly men for most measurement techniques; only cfPWV(S) and ABPI were successfully obtained in <90%, comparing well with studies in younger populations.^21–23^ Reproducibility analyses of the ultrasound assessment for cIMT and plaque were good and similar to other reported studies.^23 24^ The between visit reproducibility was also good for all measures. Comparison with other studies is difficult given the relatively large time interval between visits but despite some variations in methodology and study population age our results are comparable.^11 13 22 25^ PWV, IMT and presence of plaque all increased with age, while DC and ABPI decreased, consistent with expectations.^4 26–28^ Identification of 8.7% of men with an ABPI <0.9, indicative of obstructive PAD, is similar to a previous study.^29^ However, the proportion of men who were found to have carotid plaque was high in our population at 84%. Unsurprisingly, this was higher than other studies in slightly younger mixed populations (Rotterdam study age 69 years and Three-City Study age range 65–85 years), but more in keeping with the 71% prevalence found in an Italian study of slightly younger men and women with a mean age of 73 years.^30–32^

The lower success rate for cfPWV(S) compared with cfPWV(V) is similar to a previous study in children which used both methods to assess cfPWV.^11^ Failures in data collection mainly reflected computer failure and limited participant mobility, together with difficulties detecting a measurable pulse in specific locations. Importantly, simultaneous assessment of carotid and femoral waveforms means that the Vicorder is less affected by arrhythmias which are more prevalent in the elderly and prevented determination of cfPWV(S) in approximately 5% (74 of 1606) of our cohort. The limited success rate for recording valid ABPI data was mainly due to non-occlusion of arteries, particularly in the lower limb. This partly reflected the limit imposed on the maximum inflation pressure of the cuffs to 180 mm Hg as it was felt that pressures greater than this could have caused unacceptable discomfort to the elderly volunteers. Indeed this pressure would not have occluded the arteries of 31 men (2%) whose systolic blood pressure exceeded 180 mm Hg. In routine practice, higher cuff occlusion pressures would be applied on an individual basis where these data are required for clinical decision making.

### Comparison of methods for cfPWV assessment

Our finding of only moderate agreement (r=0.52) between the two techniques for assessment of PWV replicates that of Keehn *et al* (r=0.50), but differs from Hickson *et al* (r=0.85).^9 11^ The weaker correlation may be due in part to our large elderly population with the inherent practical and technical challenges for recording cfPWV. The level of agreement we found between the two techniques were wider than in two studies in children and adolescents but more in line with findings in a small adult population.^10 11 33^ Hickson *et al* also observed a similar bias in readings over 10 m/s.^9^

#### Path length

When investigating how different methods for deriving path length might influence the difference in cfPWV(V) and cfPWV(S) measurements, we found that using the manufacturers recommended method of path length measurement resulted in the smallest difference between the two techniques. Notably, deriving path length by the same method for each technique did not change the nature of the bias between the two techniques. Therefore, the method of path length assessment does not appear to be a major determinant of the differences in cfPWV(V) and cfPWV(S) across the cfPWV range in this population.

In order to explore the potential impact of recording separate waveforms at carotid and femoral arteries on transit times for derivation of cfPWV(S), we plotted ECG_R_ to carotid and ECG_R_ to femoral transit times separately against the Vicorder transit time. Notably, we found that the relative proportion of ECG_R_ to carotid transit time that will be subtracted from the femoral transit time to derive cfPWV(S) tends to decrease as the Vicorder transit time increases. This leads to the derivation of a relatively shorter Sphygmocor transit time (≈faster cfPWV) when cfPWV(V) is higher and a relatively longer Sphygmocor transit time (≈slower cfPWV) at lower cfPWV(V)s. This could potentially account, at least in part, for the systematic bias observed between the two methodologies across the range of cfPWVs in our cohort.

The difference in the proportion of carotid transit time subtracted from the femoral transit time at higher cfPWVs compared with lower cfPWVs required for calculation of cfPWV(S) may partly be explained by the proportion of aorta and its main branch vessels within each measurement tract. The carotid path contains proportionally less aorta than the femoral path. In early life, there is a mismatch between the elastic aorta and the more muscular vessels branching from it. This mismatch in stiffness decreases with age, as the aorta progressively stiffens to a greater extent than the more peripheral vessels.^34^ This could therefore lead to a relatively smaller change in transit time within the innominate and carotid arteries, than within the aorta over time. The impact on transit times in the ECG_R_ to carotid path would be expected to be less substantial than the changes in transit time in the ECG_R_ to femoral path given the greater proportion of aorta in the latter. Thus, an increasing proportion of carotid transit time would consequently need to be subtracted from the femoral transit time, leading to relative underestimation of aortic transit time and relatively higher cfPWV(S) values. Further studies, outside of this paper, would be required to investigate possible mechanisms, which may reflect disease processes rather than just ageing.

The different techniques used for acquiring the waveforms (applanation tonometry and cuff based volume displacement) may account for some of the discrepancies between the two measures. However, it was not within the scope of this study to investigate this. Another source of measurement bias may be related to differences in sampling rates for data collection and the number of physiological recordings required by each device. The Vicorder acquires data through two channels at 556 Hz per channel, while the Sphygmocor samples data at 128 Hz per channel using four channels giving greater potential for measurement error. Nonetheless, such measurement bias would cause PWV results to vary in both directions, as evidenced by the greater SD of cfPWV(S) vs cfPWV(V) (2.6 vs 1.7 m/s, respectively). As we observed a systematic bias whereby Sphygmocor gave higher readings at the upper end of the PWV range and Vicorder higher readings at the lower end, this cannot be accounted for by a measurement bias.

Cut-off cfPWV values have been suggested by experts in guidelines as a measure of identifying individuals at increased risk of cardiovascular events in routine clinical practice. We were interested in how the differences in results between the two methods might affect classification of risk according to cfPWV results and the consequent potential implications for cardiovascular risk management in practice. With a cut-off value of 12 m/s, more men were considered as being at increased risk with the Sphygmocor than the Vicorder. Although the numbers thus classified were similar using the lower cut-off value of 10 m/s there was still a good number of men who were considered at increased risk by one device but not the other. This does suggest that the cfPWV values for the two methods are not necessarily interchangeable and the device used for cfPWV assessment needs to be taken into account when assessing cardiovascular risk or deciding on a course of treatment. Other measures of subclinical disease assessment such as carotid intima–media thickness and/or coronary CT can of course be used in practise. Importantly, global risk scores such as QRISK and SCORE are not well validated in the elderly, for whom a more individual approach to risk management is recommended and where a simple cfPWV measurement could be a very useful addition.^35^ In the future this cohort will provide a good opportunity to directly compare the relative prognostic usage of the Vicorder and Sphygmocor data through association with hard clinical outcome in multivariate risk models. These analyses are planned for when there have been a sufficient number of major adverse cardiovascular events in this cohort to provide adequate statistical power.

### Study strength and limitations

This study was formed of a large sample of community-dwelling elderly men in which we have collected a large number of vascular measures and have been able to compare two techniques for assessing arterial stiffness. Limitations of the study are that it is based in a male European population and may have limited relevance for women or those of different ethnicity. Only 55% of the surviving men participated, those who did not attend were older and had a slightly higher BMI at a previous follow-up visit.^36^ Although this slightly limits the numbers available to study the long-term potential causal influences on vascular pathophysiology, this remains a large and unique cohort representing a relatively understudied age group. A limitation of the comparison between PWV methods is that cfPWV(S) was always assessed before cfPWV(V) as part of the larger, non-invasive arterial data collection protocol. Ideally, for such a comparison, the order of the two measures should be randomly balanced, in order to minimise the impact of any change in autonomic status during the evaluation. The consistent order of recording cfPWV with the two methods may have had an influence on the results, as the longer rest before cfPWV(V) assessment may account for some of the difference in cfPWV between methods due to lower blood pressure during cfPWV(V) assessment. Blood pressure was only assessed prior to cfPWV(S), therefore we were unable to test whether differences in blood pressure may have influenced the differences between the two methods. However, we found that the heart rate, a fairly sensitive marker of autonomic status, was similar during cfPWV(S) and cfPWV(V) recordings. This suggests that alterations in autonomic status is unlikely to have been a significant cause of the observed bias between cfPWV(S) and cfPWV(V) but its influence cannot be definitely excluded.

## Conclusion

In conclusion, we have shown the feasibility of making a detailed characterisation of the vascular phenotype in an elderly population with good success rates, tolerability and reproducibility. We have also shown that systematic differences in PWV, as assessed by the two techniques, may partly be due to the sequential measurement of carotid and femoral waveforms and subtraction of carotid transit time when calculating cfPWV(S). The measures used in this study will provide the basis for further detailed research into the understanding of cardiovascular risk and disease and clinical outcomes in the elderly.
