# Supplementary material for: Arterial pathophysiology and comparison of two devices for pulse wave velocity assessment in elderly men: the British regional heart study
Source: Open Heart. 2017 Dec 17;4(2):e000645. doi: 10.1136/openhrt-2017-000645 (PMC5761282; doi:10.1136/openhrt-2017-000645)
Supplement: Supplementary file 1 [file openhrt-2017-000645supp001.pdf]

ARTERIAL PATHOPHYSIOLOGY AND COMPARISON OF TWO DEVICES FOR PULSE WAVE VELOCITY ASSESSMENT IN ELDERLY MEN -THE BRITISH REGIONAL HEART STUDY

Supplementary Material

SUPPLEMENTARY METHODS

The British Regional Heart Study is a prospective cohort study of 7,735 men recruited when aged 40–59 years from a single primary care centre in 24 British towns in 1978–1980. 3,137 survivors were invited to undergo vascular assessment between 2010 and 2012, either at a local clinic or (if not able to attend clinic) in their own home where they underwent a limited assessment. The National Research Ethics Service (NRES) Committee London provided ethical approval. Participants provided informed written consent to the study, which was performed in accordance with the Declaration of Helsinki.

Blood pressure

Seated blood pressure was recorded from the right arm with an automated sphygmomanometer (Omron HEM 907, Japan) using an appropriately sized cuff. Trained research nurses took two measurements at 1 minute a part and the results were averaged.

Pulse Wave Velocity

Two techniques were used to assess carotid to femoral pulse wave velocity: applanation tonometry (Sphgymocor [S], Atcormedical, Australia) and an oscillometric cuff based technique (Vicorder [V], Skidmore Medical, UK). Measurements were taken with the

participant supine, with their torso at approximately 30°. cfPWV(S) was assessed first followed by cfPWV(V).

Sphygmocor cfPWV assessment

Good quality ECG-gated arterial pressure waveforms were recorded 10 seconds sequentially at the carotid and femoral arteries. Path length was calculated according to the manufacturer's recommendations, as the distance from the sternal notch (SN) to the femoral artery minus the distance from the SN to the carotid artery. These distances were measured using a standard tape measure. Transit time was calculated as the difference between the R wave of the ECG (ECG_R) to femoral pulse point and ECG_R to carotid pulse point transit times from the sequential recordings. A maximum of four recordings were made with the intention of obtaining two readings for which the standard deviation (SD) of the individual trace was ≤10 % and the difference between the two recordings was ≤0.5 m/s. If this was not achievable, results with a SD >15 % were rejected and the remaining results averaged. This measurement was not assessed during home visits.

Vicorder cfPWV assessment

A neck cuff was placed over the right carotid artery with a second cuff positioned around the middle of the right thigh. Path length was obtained using a standard tape measure to record the distance from the SN to the middle of the thigh cuff, as per manufacturer's recommendations. Transit time was derived from the simultaneous acquisition of carotid and femoral waveforms. The cuffs were simultaneously inflated to 65 mmHg. Waveforms were visually inspected and a minimum of 3 good quality waveforms were captured. Two recordings with a difference in cfPWV of ≤0.5 m/s were accepted and averaged.

Carotid Artery Ultrasound Measurements

Carotid arteries were imaged using the Z.One Ultra ultrasound system (Zonare Medical Systems, Mountain View, CA) with a 5-10-mHz linear probe. Recordings were taken in DICOM format for later offline analysis. A cross-sectional sweep from the base of the common carotid artery to the jaw bone, and longitudinal images of the common carotid artery approximately 1 cm proximal to the carotid bifurcation, were recorded. Additional images from the internal and external carotid arteries were captured if plaque was observed. Ipsilateral brachial blood pressures were taken immediately after each carotid assessment.

Carotid Intima Media Thickness (cIMT) and Distensibility Coefficient (DC)

Peak systolic and end-diastolic carotid artery diameter and cIMT (the distance between the leading edge of the intima and the media-adventitia interface) were measured from the longitudinal images using Carotid Analyser software (Medical Imaging Applications, Iowa City, IA). A region of interest of 5-10 mm was selected in a plaque free area, at least 1cm from the bifurcation. Three end-diastolic cIMT measurements were taken in both right and left carotid arteries and the mean of these measures was used. Distension for each carotid artery was calculated as the difference between 3 end-diastolic diameters and corresponding peak systolic diameter. DC for each artery was then calculated from the distension and ipsilateral blood pressure and then averaged to give overall DC.[1]

Plaque Identification

Carotid artery ultrasound scans were assessed for the presence of atherosclerotic plaques by 5 trained observers, using either the Z.One Ultra ultrasound system or Microdicom

software (Microdicom, Bulgaria). Carotid plaques were classified as a focal area of intima-media thickening ≥1.2 mm at its thickest point, or ≥50% of the adjacent IMT.[2 3]

Ankle Brachial Pressure Index

ABPI measurements were taken sequentially on both right and left sides, using the Vicorder device (Skidmore Medical, UK). Hokanson SC10 cuffs were positioned on the upper arm and lower leg (above the ankle). Photoplethysmography sensors were clipped to the end of the middle finger and the big toe. The brachial and tibial arteries were occluded simultaneously, as the cuffs were inflated to 180mmHg. As the cuffs slowly deflated, the pulse data was checked to ensure that the blood pressures were recorded at the point of the return of the pulse at both sites. Two measurements were usually made, but if the difference in sequential brachial and ankle recordings was >5mmHg three measures were taken for each side and a mean taken. Those waveforms which did not disappear during cuff inflation were marked as "non-occluding" by the observer and were subsequently excluded from the analyses.

Reproducibility Assessment

Between Visit Reproducibility of Vascular Measurements

Men from two study towns who had attended the clinic visits were asked to return for a second visit approximately one year later. 123 participants attended and underwent the identical protocol to their previous visit. Measurements were made by one of two vascular technicians on each occasion.

Reproducibility of Ultrasound Analysis

Analysis of the ultrasound images was repeated by the two observers in 109 men to assess inter-observer reproducibility of the IMT, distension and DC measurement. In addition each observer repeated their analysis of the images of 30 men for intra observer reproducibility.

The analysis of the ultrasound images for the presence of plaque was carried out by 5 observers. All observers reviewed images from the same 20 men for intra-observer reproducibility and each repeated their analysis of images for 40 men for intra-observer reproducibility.

Classification of cardiovascular risk status by cfPWV(V) and cfPWV(S) results according to expert consensus thresholds for clinical practice

Participants cardiovascular risk status was classified according to their cfPWV(V) and cfPWV(S) results using cut off values suggested by a European guidelines paper (cut off 12m/s) and a later European expert consensus paper (cut off 10 m/s).[4 5]

Statistical Data Analysis

Analyses were performed using SPSS software (Version 20.0, SPSS inc., Chicago, Illinois). Data is presented as mean ± SD or 95% CI unless otherwise stated. Variability of the vascular measures was assessed by coefficient of variation (CV%). The exceptions to this were the identification of the presence plaque which used Gwet's Agreement Coefficient 1(AC1), and ABPI which was assessed using intra-class correlation (ICC).[6]

Differences between the two PWV methods were investigated using paired t-tests and Bland-Altman plots.[7] Correlations were examined using Pearson coefficients. Linear regression was used to investigate the relationship between difference in PWV and the

mean PWV of the two methods; models were adjusted for age and systolic and diastolic blood pressure, heart rate, height, weight and body mass index (BMI) as individual variables. A final model included age, diastolic blood pressure, heart rate and BMI simultaneously entered into the model.

SUPPLEMENTARY RESULTS

Missing ABPI and PWV data

In 323 participants it was not possible to obtain an ABPI measurement from either side. Of these men, 196 were reported as having non-occluding traces; 31 of these men had brachial sBP greater than the maximum cuff inflation pressure of 180mmHg; 165 of these men had brachial sBP <180mmHg and therefore likely to have poorly compressible arteries in the lower limb. Technical difficulties accounted for the missing data in the remaining 127 men. For cfPWV measurements were not obtainable due to computer failure (S=27, V=7), inaccessible femoral pulse (e.g. immobility/colostomy bag/catheter/ incontinence underwear, S=145, V=47), poor quality pulse waveforms (S=21, V=20), pulse irregularity (S=74, V=0), inadequate quality ECG (S=23, V=0) and unclassified reasons (S=5 V=1).

Differences in path length

To investigate the differences between the two measures of PWV we looked at whether the different methods of determining path length were responsible. Although path length was longer for the Vicorder measurements as expected (mean difference -270 mm [95%CI-272 - -268]) Bland-Altman analysis excluded systematic bias between the two methods (supplementary figure 2).

To examine whether the difference in approach to path length estimation might explain the difference in cfPWV(V) and cfPWV(S), PWV was recalculated in a subgroup of the cohort (n=189). When cfPWV(S) was recalculated using the method for measuring Vicorder path length, (distance between the SN and femoral measurement point), the value increased from 10.38 m/s (SD 2.71) to 12.61 m/s (SD 3.11). In addition the difference between

cfPWV(S) and cfPWV(V) increased from 0.56 m/s (95%CI 0.25-0.88) to 2.79 m/s (95%CI 2.41-3.18). This change in approach did not modify the bias between the two methods (supplementary figure3A).

When VPWV was recalculated using the Sphygmocor path length method, (subtracting the SN to carotid cuff distance from the SN to femoral distance), the value decreased from 9.81 m/s (SD 1.49) to 8.98 m/s (SD 1.40). This remained lower than the original cfPWV(S) in this subset (10.38 m/s [SD 2.71] $p<0.001$). The difference between cfPWV(V) and cfPWV(S) was 1.40 m/s (95%CI 1.08-1.71), which remained greater than the primary comparison, with the bias still apparent (supplementary figure 3B).

cfPWV(S)and cfPWV(V) were then both recalculated using the path length method recommended in a recent Expert consensus paper (distance between the carotid and femoral measurement points multiplied by 0.8)[5]. Using this path length method, cfPWV(S) increased to 11.87 m/s (SD 3.04), whilst cfPWV(V) decreased to 8.50 m/s (SD 1.28). Furthermore, the difference between the two measures increased to 3.37 m/s (95%CI 3.00 – 3.73). The bias between the two measures remained (supplementary figure 3C).

SUPPLEMENTARY TABLES

Supplementary table 1: Participant characteristics for those with cfPWV data using both techniques, cfPWV(V) only and cfPWV(S) only.

	**Both PWV (1122)**	**Only cfPWV(V) (359)**	**Only cfPWV(S) (57)**
**Age**	78.0 ± 4.4	79.3 ± 5.0[a]	78.4 ± 4.2
**Systolic Blood Pressure (mmHg)**	147 ± 19	141 ± 20[a]	144 ± 21
**Diastolic Blood Pressure (mmHg)**	76 ± 11	76 ± 12	74 ± 10
**Heart rate (bpm)**	65 ± 12	73 ± 16[a]	64 ± 10
**Height (cm)**	171.6 ± 6.4	170.8 ± 6.6 [a]	170.5 ± 5.9
**Weight (kg)**	78.3 ± 11.4	83.3 ± 14.6 [a]	80.3 ± 12.4
**BMI (kg/m^2)**	26.5 ± 3.3	28.5 ± 4.5 [a]	27.6 ± 3.8 [a]
**cfPWV(V)**	10.1 ± 1.7	10.5 ± 1.7[a]	
**cfPWV(S)**	10.3 ± 2.6		9.7 ± 2.5

Data presented as mean ± SD. [a] = $p<0.05$ when compared with both PWV values. BMI, body mass index; cf, carotid to femoral; PWV, pulse wave velocity; S, Sphygmocor; V, Vicorder.

Supplementary table 2: Linear regression analyses looking at the association between mean S & V cfPWV and differences in S & V cfPWV and the influence of risk factors

A

Association between mean cfPWV and differences in $cfPWV_{S-V}$ adjusted for risk factors				
	B[≠]	SE	95%CI	p
**Unadjusted**	0.56	0.03	0.50,0.62	<0.001
**Age (yrs)**	0.59	0.03	0.53,0.66	<0.001
**sBP*(mmHg)**	0.61	0.03	0.55,0.68	<0.001
**dBP*(mmHg)**	0.61	0.03	0.55,0.68	<0.001
**HR* (bpm)**	0.60	0.03	0.53,0.66	<0.001
**Height* (cm)**	0.60	0.03	0.53,0.66	<0.001
**Weight* (kg)**	0.58	0.03	0.52,0.65	<0.001
**BMI*(kg/m^2)**	0.58	0.03	0.52,0.65	<0.001
**Model 1**	0.60	0.03	0.54,0.67	<0.001

B

Influence of individual risk factors on relationship between mean cfPWV and differences in $cfPWV_{S-V}$								
	**Each risk factor adjusted for age**				**Model 1**			
	**B°**	**SE**	**95%CI**	**p**	**B°**	**SE**	**95%CI**	**p**
**Age (yrs)**	-0.06	0.01	-0.087,-0.033	<0.001	-0.06	0.01	-0.086,-0.030	<0.001
**sBP*(mmHg)**	-0.01	0.003	-0.013,0.003	0.06				
**dBP*(mmHg)**	-0.02	0.01	-0.026,-0.005	0.005	-0.01	0.01	-0.025,-0.004	0.009
**HR*(bpm)**	-0.003	0.005	-0.012,0.007	0.58	0.00	0.01	-0.009,0.010	0.91
**Height*(cm)**	-0.013	0.009	-0.030,0.010	0.16				
**Weight*(kg)**	0.014	0.005	0.005,0.020	0.007				
**BMI*(kg/m^2)**	0.073	0.018	0.040,0.110	<0.001	0.07	0.02	0.036,0.107	<0.001

A; following adjustment for individual risk factors with age and a model (model 1) including age, diastolic blood pressure, heart rate and BMI **B**; the effect size of each risk factor following individual adjustment with age and those variables included model 1. * adjusted for age; SBP, systolic blood pressure; DBP, diastolic blood pressure; HR, heart rate; BMI, body mass index; B, regression coefficients; SE, standard error; CI, confidence intervals. [≠]Regression coefficient shows change in differences in S&V cfPWV for 1 unit change in mean S&V cfPWV after adjustment for individual risk factors. °Regression coefficient shows change in differences in S&V cfPWV for 1 unit change in the risk factor.

SUPPLEMENTARY FIGURES

Supplementary figure 1: Vascular measures by age group. **A** Pulse wave velocity (PWV) Sphygmocor, **B** Pulse wave velocity (PWV) Vicorder, **C** Mean carotid intima-media thickness (cIMT), **D** Prevalence of plaque **E** Carotid Distensibility Coefficient (DC) and **F** Mean ankle brachial pressure index (ABPI) by age group. Data are Mean ± S.D or percentage.

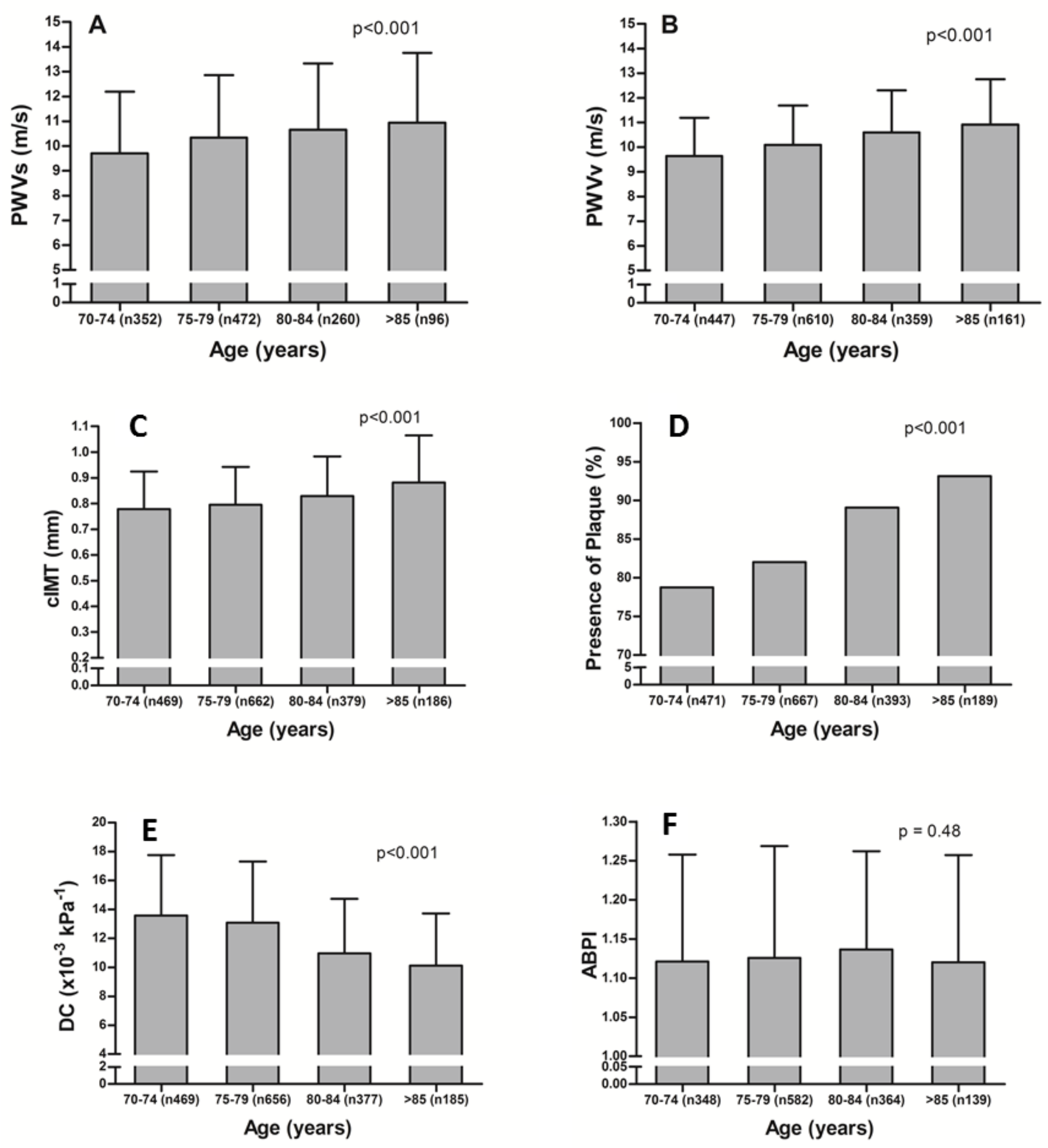

Supplementary figure 2: Bland-Altman plots of agreement between Sphygmocor path length and Vicorder path length

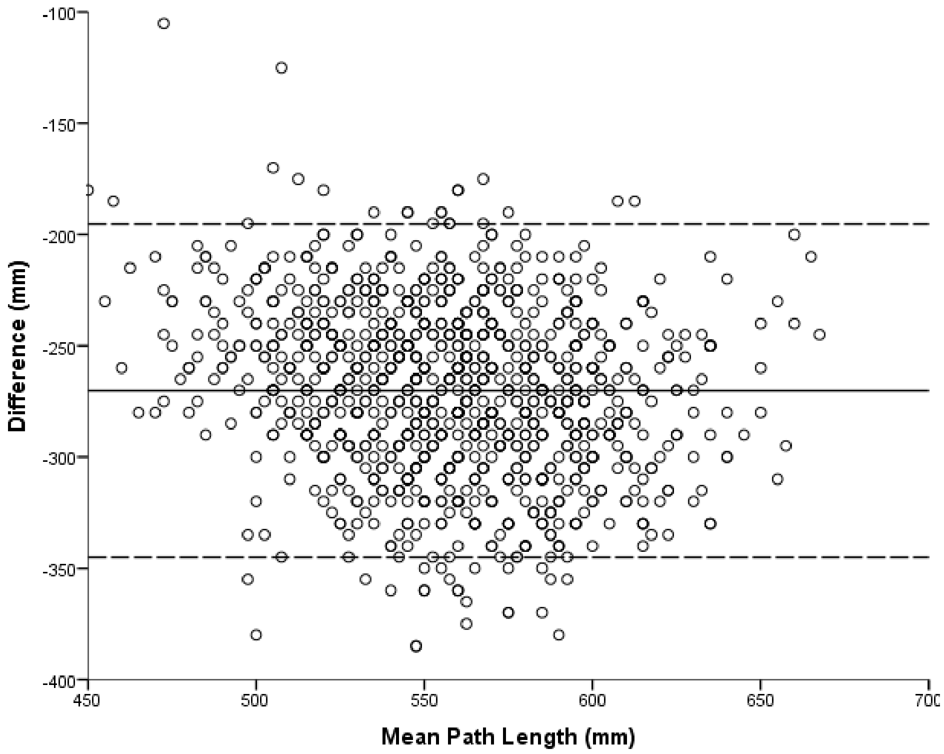

Supplementary figure 3: Bland-Altman plots of agreement between **A,** cfPWV (S)with modified path length and cfPWV(V) measured according the manufacturers protocol; **B,** cfPWV(S) path lengh measured according to the manufacturers protocol and cfPWV(V) with modified path length; C, path length for both cfPWV(S) and cfPWV(V) measured according to method recommended in recent Expert consensus paper[5]

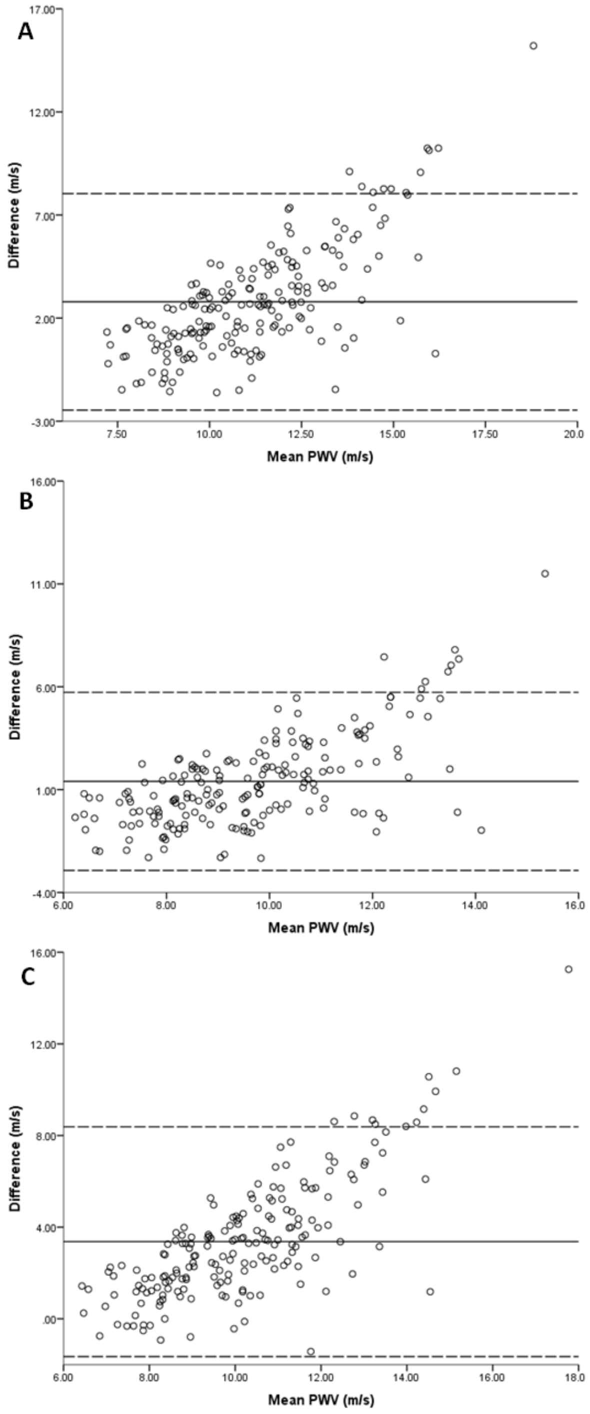

SUPPLEMENTARY REFERENCES

1. Dijk JM, Algra A, van der GY, et al. Carotid stiffness and the risk of new vascular events in patients with manifest cardiovascular disease. The SMART study. *European Heart Journal* 2005;**26**(12):1213-20.
2. Ebrahim S, Papacosta O, Whincup P, et al. Carotid Plaque, Intima Media Thickness, Cardiovascular Risk Factors, and Prevalent Cardiovascular Disease in Men and Women : The British Regional Heart Study. *Stroke* 1999;**30**(4):841-50.
3. Touboul PJ, Hennerici MG, Meairs S, et al. Mannheim carotid intima-media thickness consensus (2004-2006). An update on behalf of the Advisory Board of the 3rd and 4th Watching the Risk Symposium, 13th and 15th European Stroke Conferences, Mannheim, Germany, 2004, and Brussels, Belgium, 2006. *CerebrovascDis* 2007;**23**(1):75-80.
4. Mancia G, De Backer G, Dominiczak A, et al. 2007 Guidelines for the Management of Arterial Hypertension: The Task Force for the Management of Arterial Hypertension of the European Society of Hypertension (ESH) and of the European Society of Cardiology (ESC). *Journal of Hypertension* 2007;**25**(6):1105-87.
5. Van Bortel LM, Laurent S, Boutouyrie P, et al. Expert consensus document on the measurement of aortic stiffness in daily practice using carotid-femoral pulse wave velocity. *Journal of Hypertension* 2012;**30**(3):445-48.
6. Gwet KL. Computing inter-rater reliability and its variance in the presence of high agreement. *British Journal of Mathematical and Statistical Psychology* 2008;**61**(1):29-48.
7. Bland JM, Altman DG. Statistical methods for assessing agreement between two methods of clinical measurement. *Lancet* 1986;**1**(8476):307-10.
